# Transcriptomic and Expression Analysis of the Salivary Glands in White-Backed Planthoppers, *Sogatella furcifera*

**DOI:** 10.1371/journal.pone.0159393

**Published:** 2016-07-14

**Authors:** Zhen Li, Xing-Kui An, Yu-Di Liu, Mao-Lin Hou

**Affiliations:** State Key Laboratory for Biology of Plant Diseases and Insect Pests, Institute of Plant Protection, Chinese Academy of Agricultural Sciences, No. 2, West Yuan Ming Yuan Road, Beijing 100193, China; Institute of Vegetables and Flowers, Chinese Academy of Agricultural Sciences, CHINA

## Abstract

The white-backed planthopper (WBPH), *Sogatella furcifera* (Horváth), is one of the serious rice pests because of its destructive feeding. The salivary glands of the WBPH play an important role in the feeding behaviour. Currently, however, very little is known about the salivary glands at the molecular level. We sequenced the salivary gland transcriptome (sialotranscripome) of adult WBPHs using the Illumina sequencing. A total of 65,595 transcripts and 51,842 unigenes were obtained from salivary glands. According to annotations against the Nr database, many of the unigenes identified were associated with the most studied enzymes in hemipteran saliva. In the present study, we identified 32 salivary protein genes from the WBPH sialotranscripome, which were categorized as those involved in sugar metabolism, detoxification, suppression of plant defense responses, immunity-related responses, general digestion, and other phytophagy processes. Tissue expression profiles analysis revealed that four of 32 salivary protein genes (multicopper oxidase 4, multicopper oxidase 6, carboxylesterase and uridine phosphorylase 1 isform X2) were primarily expressed in the salivary gland, suggesting that they played putative role in insect-rice interactions. 13 of 32 salivary protein genes were primarily expressed in gut, which might play putative role in digestive and detoxify mechanism. Development expression profiles analysis revealed that the expression level of 26 of 32 salivary protein genes had no significant difference, suggesting that they may play roles in every developmental stages of salivary gland of WBPH. The other six genes have a high expression level in the salivary gland of adult. 31 of 32 genes (except putative acetylcholinesterase 1) have no significant difference in male and female adult, suggesting that their expression level have no difference between sexes. This report analysis of the sialotranscripome for the WBPH, and the transcriptome provides a foundational list of the genes involved in feeding. Our data will be useful to investigate the mechanisms of interaction between the WBPH and the host plant.

## Introduction

The saliva of insect herbivores contains a diversity of digestive enzymes and components, which either induce or inhibit plant defence [1]. Therefore, as the first substance to contact the plant, herbivore saliva plays an important role in the ingestion of food and in the interaction between plant and herbivore [2]. Hemipterans are phloem feeders with piercing-sucking mouthparts. The salivary organs of hemipterans are a pair of primary and accessory salivary glands, which produce two primary types of saliva: coagulable and watery [3, 4]. During feeding, they discharge the gelling and watery saliva into the rice plant tissues. Furthermore, most hemipteran vectors secrete and inoculate pathogens into healthy plants through the proteins of saliva [5, 6]. Thus, the saliva of phloem feeders is a mediator of plant-(pathogen)-insect interactions [5, 6].

The white-backed plant hoppers (WBPH; *Sogatella furcifera*; Hemiptera: Delphacidae), which is one of the most destructive insect pests of rice (*Oryza sativa* L.) in Asia. Adults and nymphs cause rice physiological abnormalities through the mouth to suck rice phloem sap, seriously lead to the plants die. WBPH is also the vector of southern rice black-streaked dwarf virus (SRBSDV) [7, 8], which outbreaks in 2009 not only in the southern Chinese province but also in northern Vietnam, and Japan [9].

Despite their importance as pests, little is currently known about the secretion and composition of WBPH saliva. Because of the important functions of herbivore saliva in plant-insect interactions, the saliva of WBPHs is likely to play a central role in the interaction between insect and rice. Therefore, information on salivary secretions is crucial for understanding the interactions between WBPHs and host plants, and the characterisation of WBPH saliva will provide new insights into WBPH-rice interactions, including induced defences in rice and WBPHs. Additionally, the results will facilitate the development of better strategies for pest control.

The transcriptomes of the salivary glands of phloem-sap feeders were determined for several hemipterans, including the rice brown plant hopper (*Nilaparvata lugens* (Stål); Hemiptera: Delphacidae) [2], the small brown planthopper (*Laodelphax striatellus* (Fallén); Hemiptera: Delphacidae) [10], the whitefly (*Bemisia tabaci* (Gennadius); Hemiptera: Aleyrodidae) [5], the potato leafhopper (*Empoasca fabae* (Harris); Hemiptera: Cicadellidae) and the green rice leafhopper (*Nephotettix cincticeps* (Uhler); Hemiptera: Cicadellidae) [11, 12]. Many proteins in the saliva (including secretory proteins) were found. Sharma et al. (2014) [4] divided the salivary proteins of plant-feeding hemipteroids into three categories according to the function: ‘detoxifying plant alleochemicals and altering plant-defence mechanism’ (e.g., glucose dehydrogenase, glucose oxidase, phenol oxidases, laccase, peroxidases, catalase, trehalases); ‘plant-cell degrading’ (e.g., *α*-glucosidases, amylase, sucrase, acid, alkaline phosphatases and peptidases); ‘other proteins’ (e.g., calcium-binding proteins, effector proteins and other non-enzymatic proteins). Glucose dehydrogenase was identified to detoxify plant defensive compounds in Russian Wheat Aphid (*Diuraphis noxia*) [13]. The phylogenetic relationships and expression patterns of multicopper oxidase gene family was first analysed to elucidate their functions in the context of *N*. *lugens* growth and development [14]. The tissue-, development-, and sex-specific expressions of serine protease gene family in *N*. *lugens* were clarified the potentially functional roles in the biological process [15]. Angiotensin converting enzyme was considered as a potential target for development of insect growth regulators [16]. Soluble and membrane-alkaline phosphatases were identified in *N*. *lugens*, and soluble-alkaline phosphatases may play a role in development and insecticide tolerance [17]. Levels of soluble and membrane-bound trehalases expression in various stages and tissues of *B*. *tabaci* suggested that soluble trehalases may prevent trehalose in salivary gland from leaking and entering into plants along with saliva, while membrane-bound trehalases may play role in trehalose catabolism during development [18].

Although many salivary proteins were identified in the hemipterods, the salivary components of WBPH and the functions of these different components are unknown. Because the complete genome of the WBPH is not available yet, a more effective method is required for transcriptomic analysis of the salivary glands of the WBPH. The sialotranscripome of WBPH was sequenced on Illumina sequencing platform with depth of 14.58 G, which can dig into more genes and assemble more long and accurate sequences.

In this study, the WBPH sialotranscripome was sequenced and assembled, and analysis of sialotranscripome could get accurate sequences of annotated genes and reveal gene pathways. We identified the tissue and development expression of 32 salivary protein genes in WBPH. Based on this study, a rich molecular resource for future functional studies on primary salivary glands can be provided, which will contribute to a better understanding of the insect-rice interactions.

## Methods

### Insect rearing and salivary glands collection

The original WBPH adults used in the experiment were collected separately from Xing'an County, Guilin City, Guangxi Zhuang Autonomous Region, China, in 2013, which is the Scientific Observing and Experimental Station of Crop Pests of Guilin/Guilin Branch, Institute of Plant Protection, Chinese Academy of Agricultural Sciences. The field studies did not involve endangered or protected species, and no specific permissions were required for these activities in this station. After collection, the planthoppers were then maintained artificially in our laboratory. The WBPHs cultured on TN-1 rice plants were reared in an artificial climatic chamber at 26 ± 1°C and 70 ± 10% relative humidity under a 16/8 h light/dark photoperiod.

Salivary glands (approximately 600 of each sex) were collected from 1- to 3-day-old adults for transcriptome sequencing. The adult WBPHs were anesthetised with ice and placed in a petri dish that was also on ice. The salivary glands of WBPHs were dissected in a drop of sterilised 1× Phosphate Buffered Saline (1× PBS) solution with microforceps (Shanghai Medical Instruments Ltd., Corp.) under an anatomical lens (Leica Microsystems GmbH, Wetzlar, Germany). The head was first pulled from the pronotum, and then the pair of salivary glands that emerged at the distal end of the severed head was carefully removed with microforceps. Different tissues including salivary gland (SG), head (without salivary gland), gut, malpighian tubule (MT), and remaining body (RB) of adult WBPHs and salivary gland at different developmental stages (2^nd^-3^rd^ instar, 4^th^-5^th^ instar, female and male adult) of WBPHs were collected for real-time quantitative PCR (qPCR) test. All collected tissues were immediately frozen and stored at -80°C until use.

### Library construction of the salivary glands and Illumina sequencing

Approximately 300 female and 300 male salivary glands were pooled together for one sample. Total RNA was extracted using the PureLink RNA Mini Kit (Life technologies, Carlsbad, CA, USA) according to the manufacturer’s instructions. The degradation and contamination of RNA sample was monitored on 1% agarose gels, and the RNA purity was checked using the NanoPhotometer^®^ spectrophotometer (IMPLEN, CA, USA), RNA concentration was measured using Qubit^®^ RNA Assay Kit in Qubit^®^ 2.0 Flurometer (Life Technologies, Carlsbad, CA, USA), and the RNA integrity was assessed using the RNA Nano 6000 Assay Kit of the Agilent Bioanalyzer 2100 system (Agilent Technologies, CA, USA).

Messenger RNAs were purified from total RNA using poly-T oligos attached to magnetic beads. The mRNAs were then shared into short fragments using fragmentation buffer. First strand cDNA were synthesized using random hexamer primer and M-MuLV Reverse Transcriptase (RNase H^-^). Second strand cDNA synthesis was subsequently performed using DNA Polymerase I and RNase H. Remaining overhangs were converted into blunt ends via exonuclease/polymerase activities. After adenylation of 3’ ends of DNA fragments, NEBNext adaptor with hairpin loop structure were ligated to prepare for hybridization. In order to select cDNA fragments of preferentially 150~200 bp in length, the library fragments were purified with AMPure XP system (Beckman Coulter, Beverly, USA). Then 3 μl USER Enzyme (NEB, USA) was used with size-selected, adaptor-ligated cDNA at 37°C for 15 min followed by 5 min at 95°C before PCR. Then PCR was performed with Phusion High-Fidelity DNA polymerase, Universal PCR primers and Index (X) Primer. At last, PCR products were purified (AMPure XP system) and library quality was assessed on the Agilent Bioanalyzer 2100 system. The clustering of the index-coded samples was performed on a cBotCluster Generation System using TruSeq PE Cluster Kit v3-cBot-HS (Illumina) according to the manufacturer’s instructions. After cluster generation, the library preparations were sequenced on an Illumina Hiseq platform and paired-end reads were generated.

### Bioinformatics data analysis

Raw reads of fastq format were firstly processed through in-house perl scripts. In this step, clean reads were obtained by removing low quality reads and reads containing adapter, reads containing ploy-N. At the same time, Q20, Q30, GC-content and sequence duplication level of the clean data were calculated. Transcriptome assembly based on clean reads with high quality was accomplished using Trinity [19]. Following the assembly, homology searches of all unigenes were conducted using the BLASTx and BLASTn programs against Nr (non-redundant protein database) and Nt (nucleotide sequence databases) in NCBI with an E-value less than 1.0E-5. Unigenes were also aligned by BLASTx with other protein databases, including Gene Ontology (GO), Swiss-Prot, Protein Family (PFAM), Kyoto Encyclopedia of Genes and Genomes (KEGG) ontology (KO), and euKaryotic Ortholog Groups (KOG), to identify protein with high sequence similarity and assign putative functional annotations. By using Blast2GO program [20], GO terms were extracted from the best hits obtained from the BLASTx against the Nr and PFAM.

### Identification of putative WBPH Salivary protein genes

Salivary protein genes which were categorized as those involved in sugar metabolism, detoxification, suppression of plant defense responses, immunity-related responses, general digestion, and other phytophagy processes were selected in the sialotranscripome annotation of WBPH. The longest open reading frame (ORF) of each unigene was determined using the ExPASY Translate Tool (http://web.expasy.org/translate/). The sequences of all candidate salivary protein were checked on the BLASTx program at the NCBI by manually.

### Verification of the salivary protein sequences by cloning and sequencing

Gene-specific primers designed by Primer 5.0 program were used to clone the ORF or partial sequences of each salivary protein gene (see Supplementary Material: S1 Table). The total RNA from the whole adult was extracted by using RNAiso plus Reagent (TaKaRa, Dalian, China) and template cDNA was synthesized using the Fast Quant RT kit (TIANGEN, Beijing, China). Each PCR reaction (25 μl volume) was carried out with 200 ng cDNA and the cycling conditions were set at 95°C for 3 min with 35 cycles of 94°C for 30 sec, 57°C for 1 min, 72°C for 1 min, and a final extension at 72°C for 5 min. The PCR products were gel-purified and cloned into the pGEM^®^-T Easy Vector (Promega Corporation, Madison, USA), and the insert was sequenced with standard M13 primers. Then all sequences of candidate salivary protein genes were manually checked by the BLASTx program at the NCBI.

### Real-time qPCR measurement

The relative transcript abundance of salivary protein genes in different tissues (SG, head, gut, MT, and RB) and in the salivary gland of different developmental stages and sexes (2^nd^-3^rd^ instar, 4^th^-5^th^ instar, female adult, male adult) were analysed by qPCR using an ABI 7500 Real-Time PCR System (Applied Biosystems, Carlsbad, CA, USA). Due to the tissues (SG, gut, MT) were infinitesimal, total RNA was extracted by using the PureLink RNA Mini Kit (Life technologies, Carlsbad, CA, USA) according to the manufacturer’s instructions. Total RNA of other body parts (head and RB) was extracted by using RNAiso plus Reagent (TaKaRa, Dalian, China). For each total RNA sample, 0.6 μg of RNA was reverse-transcribed to cDNAs by the Fast Quant RT Kit (TIANGEN, Beijing, China). Two references genes Ribosomal protein L9 (GeneBank Acc. KP735523) and Ribosomal protein L10 (GeneBank Acc. KP735524) were used for normalization, as they were stably expressed at different tissues and development stages [21]. The primers of target and reference genes were designed with the program primer3 web (version 4.0.0) (http://primer3.ut.ee/) and listed in Supplementary Material: S2 Table. The specificity and efficiency of each primer was validated by analysing standard curves with a five-fold cDNA dilution series. Each qPCR reaction was conducted in a 20 μl mixture containing 10 μl of Bester^®^ SybrGreen qPCR mastermix (DBI^®^ Bioscience, Germany), 0.4 μl of each primer (10 μM), 0.04 μl of 50 × ROX Reference Dye, 4 μl of sample cDNA, and 5.16 μl of sterilized H_2_O. The qPCR cycling parameters were as follows: 95°C for 5 min, followed by 40 cycles of 95°C for 10 sec and 60°C for 31 sec, melt curves stages at 95°C for 15 sec, 60°C for 1 min, and 95°C for 15 sec. Negative controls without template were included in each experiment. To check reproducibility, the qPCR reaction for each sample was performed in three technical replicates and two biological replicates. The comparative 2^−ΔΔCT^ method [22] was used to calculate the relative quantification between tissues and salivary glands in development stages and sexes of WBPH. Data analysis was performed using the SPSS Statistics 19.0 software (IBM SPSS Statistics Inc., Chicago, IL, USA). A one-way nested analysis of variance (ANOVA) and Duncan’s new multiple range test (*p* < 0.05) were used to calculate the relative expression of each target gene. The values were presented as the mean ± SE when applicable.

## Results

### Illumina HiSeq sequencing and reads assembly

The cDNA libraries of salivary gland of WBPH were sequenced with Illumina HiSeq. A total of 99,820,374 raw reads were produced from salivary gland samples. After trimming the adaptor sequences and removing low quality sequences, 97,174,264 clean reads were remained for following assembly and produced 65,595 (mean length 874 bp) transcripts, respectively. The longest transcript of each gene is a unigene, and the number of unigenes was 51,842 (mean length 746 bp) (Table 1). The transcriptome raw reads had been deposited with NCBI SRA database (accession number: SRR3211109).

**Table 1:** An overview of sequencing and assembly process.

Total size	14.58 G
Total number of raw reads	99,820,374
Total number of clean reads	97,174,264
Total number of transcripts	65,595
Transcripts mean length	874 bp
Total number of unigenes	51,842
Unigenes mean length	746 bp

### Functional annotation of the WBPH salivary gland unigenes

**Fig 1:**
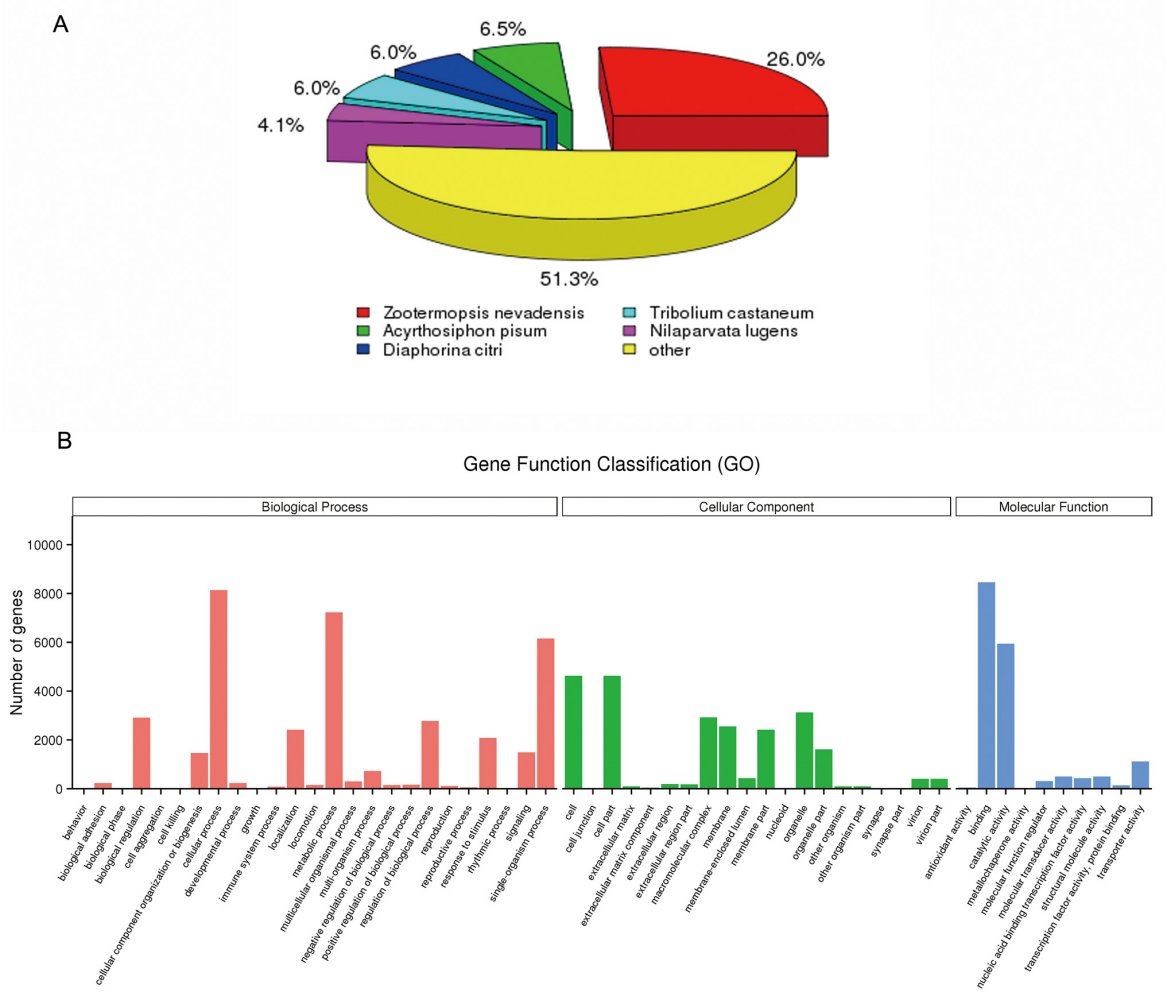
Summary for the annotation of WBPH salivary gland transcriptome. (A) Species distribution of best BLASTx hits of salivary gland transcriptome. (B) Gene Ontology (GO) classifications of WBPH salivary gland unigenes according to their involement in biological process, cellular component and molecular function.

In total, all of 51,842 unigenes matched known proteins within all databases, through using Nr, Nt, KO, SwissProt, PFAM, GO and KOG. Through annotation by BLASTx and BLASTn program with the E-value cut-off of 1.0E-5, 17,767 of the 51,842 unigenes (34.27%) had BLASTx hits in the Nr databases and 4,068 (7.84%) had BLASTn hits in the Nt databases. The species distributions of the best BLASTx hit for each sequence are shown in Fig 1A. The highest percentage of unigene sequences were matched with *Zootermopsis nevadensis* (26.0%), followed by *Acyrthosiphon pisum* (6.5%), *Diaphorina citri* (6.0%), *Tribolium castaneum* (6.0%) and *N*. *lugens* (4.1%). A similar pattern of highest similarity with *T*. *castaneum* in the BLAST annotation was also reported for the transcriptomes of *S*. *furcifera*, *N*. *lugens* and *N*. *lugens* salivary glands, with a similarity of 16.17%, 18.89% and 14.34% with *T*. *castaneum*, respectively [2, 23, 24].

**Fig 2:**
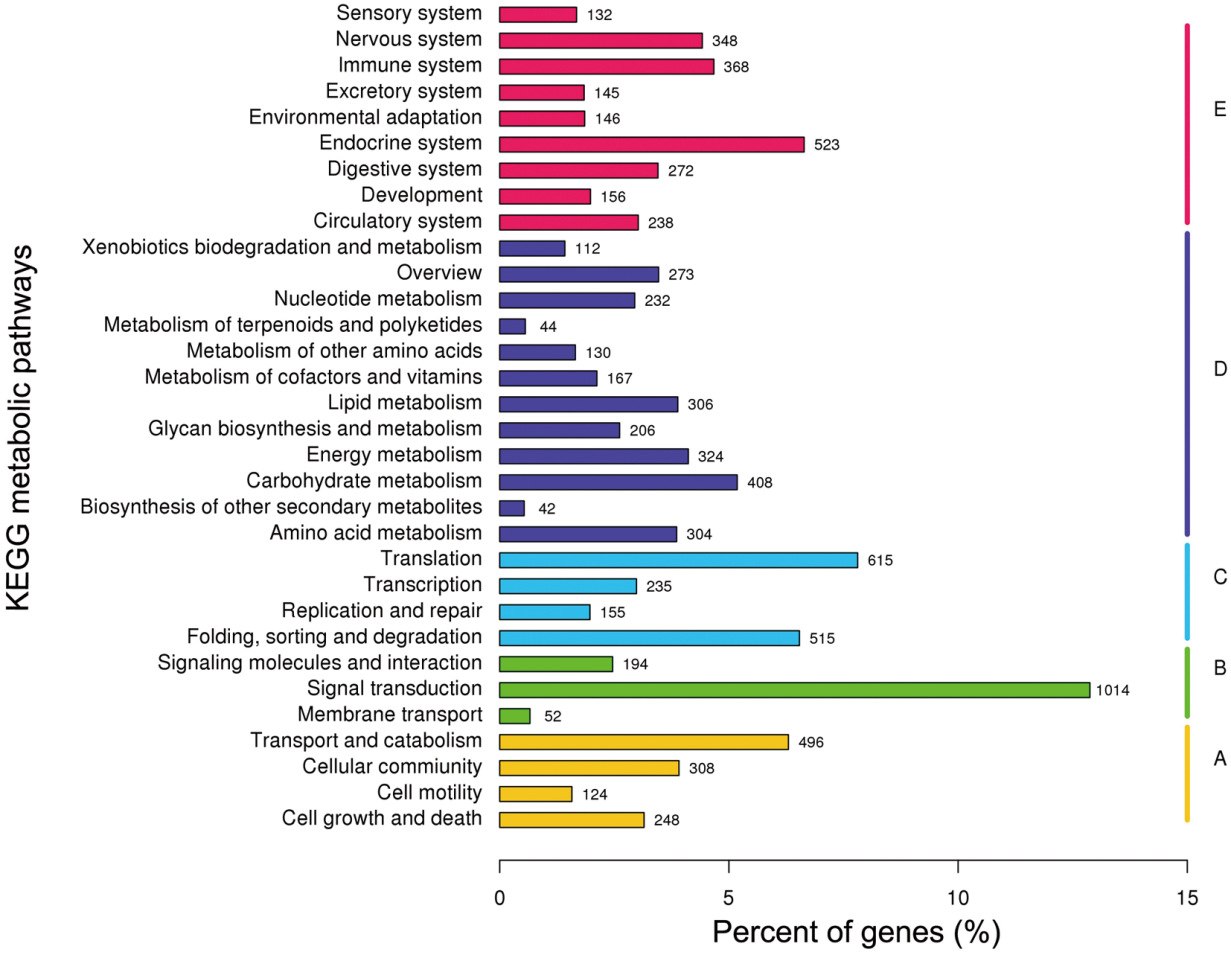
KEGG pathway distributions of WBPH salivary gland unigenes. The genes according to KEGG metabolic pathway involved was divided into five branches: A. Cellular processes; B. Environmental information processing; C. Genetic information processing; D. Metabolism; E. Organismal systems.

GO assignments were used to functionally classify the predicted proteins. The GO terms were functionally grouped into three primary divisions: ‘biological process,’ ‘molecular function’ and ‘cellular component.’ Among the 51,824 unigenes, 14,753 (28.45%) could be assigned to various GO terms and annotated, and they were categorised into 55 functional groups (Fig 1B). To investigate the biological pathways that were active in the salivary glands, the sequences were assigned to the reference canonical pathways in the KEGG (Fig 2). A total of 7,876 unigenes were mapped separately to a total of 261 pathways, the top 32 of which were depicted in Fig 2. Among these pathways, the largest number of sequences (2,548 unigenes) was involved in ‘metabolic pathways’, which confirmed that metabolism is important in the salivary glands. The GO annotations and KO classifications indicated that the salivary glands were active in metabolism, binding and signal transduction.

### Identification of WBPH salivary proteins

We identified 32 putative salivary protein genes in the WBPH Illumina HiSeq sialotranscripome data (Table 2). 24 of 32 putative salivary protein genes (except serine protease snake-4, serine protease snake-6, serine protease-11, cytochrome P450 CYPSF04, putative acetylcholinesterase 1, prostatic acid phosphatase, calcium/calmodulin-dependent protein kinase 2 isform X2 and calcium/calmodulin-dependent protein kinase type 1 isoform X3) have intact ORF with lengths ranging from 1050 bp to 2805 bp. Membrane-bound trehalase (GeneBank Acc. AFO54713.1) and NADPH-cytochrome P450 reductase (GeneBank Acc. AHM93009.1) have been identified in WBPH. All of the 32 salivary proteins were manually checked by the BLASTx program and then named according to the highest protein similarities with the high amino acid identities (48%–100%) of the best BLASTx hit at NCBI. The nucleotide sequences of the 32 salivary protein genes were confirmed by cloning and sequencing, and then deposited in the GenBank under the accession numbers KU764420 to KU764449 except membrane-bound trehalase and NADPH-cytochrome P450 reductase, which were further aligned to analysis and found to hit 100% with the deposited ones.

**Table 2:** Genes of interest identified in the *S*. *furcifera* sialotranscriptome.

Gene name	Accession number	Query length (bp)	ORF (aa)	Complete-ness	Blastx annotation	Score	*E*-value	% Identify
**Sugar metabolism**								
Alpha-glucosidase family 31	KU764420	2630	677	Complete	gb|AIA09350.1|Alpha-glucosidase family 31, partial [*Periplaneta americana*]	578	0	46%
Neutral alpha-glucosidase AB	KU764421	3687	935	Complete	gb|KDR15365.1|Neutral alpha-glucosidase AB [*Zootermopsis nevadensis*]	1144	0	59%
Lysosomal alpha-glucosidase	KU764423	3885	926	Complete	gb|KDR14932.1|Lysosomal alpha-glucosidase [*Zootermopsis nevadensis*]	953	0	51%
Soluble trehalase	KU764425	2813	575	Complete	gb|AFL03409.1|soluble trehalase [*Laodelphax striatella*]	1092	0	95%
Membrane-bound trehalase	AFO54713.1	5052	613	Complete	gb|AFO54713.1|membrane-bound trehalase [*Sogatella furcifera*]	1199	0	99%
UDP-N-acetylglucosamine pyrophosphorylase	KU764442	3166	489	Complete	gb|AEL88647.1|UDP-N-acetylglucosamine pyrophosphorylase [*Nilaparvata lugens*]	751	0	97%
**Detoxification and inhibition of plant defenses**								
Glucose dehydrogenase [acceptor]	KU764422	5499	624	Complete	gb|KDR15959.1|Glucose dehydrogenase [acceptor], partial [*Zootermopsis nevadensis*]	1123	0	52%
Alkaline phosphatase-like isoform X1	KU764424	2068	524	Complete	ref|XP_012278502.1|PREDICTED: alkaline phosphatase-like isoform X1 [*Orussus abietinus*]	544	0	53%
Multicopper oxidase 4	KU764431	2965	721	Complete	gb|AKN21382.1|multicopper oxidase 4 [*Nilaparvata lugens*]	1174	0	79%
Multicopper oxidase 6	KU764432	3214	630	Complete	gb|AKN21384.1|multicopper oxidase 6 [*Nilaparvata lugens*]	1213	0	91%
Catalase	KU764433	1860	503	Complete	emb|CCO56224.1|catalase [*Nilaparvata lugens*]	978	0	95%
Cytochrome P450 SF	KU764434	2408	540	Complete	gb|AGN52753.1|cytochrome P450 [*Laodelphax striatella*]	901	0	85%
Cytochrome P450 CYPSF01	KU764435	1960	546	Complete	gb|AFU86439.1|cytochrome P450 CYP6FJ1v2 [*Laodelphax striatella*]	971	0	87%
Cytochrome P450 CYPSF02	KU764436	2120	503	Complete	gb|AIW79993.1|cytochrome P450 CYP427A1 [*Nilaparvata lugens*]	896	0	83%
Cytochrome P450 CYPSF03	KU764437	2403	519	Complete	gb|AFU86445.1|cytochrome P450 CYP6BD10v2 [*Laodelphax striatella*]	1020	0	93%
Cytochrome P450 CYPSF04	KU764438	1229	307	partial	gb|ACX54783.1|cytochrome P450 CYP3A25 [*Nilaparvata lugens*] partial	502	7e-173	87%
NADPH-cytochrome P450 reductase	AHM93009.1	3613	677	Complete	gb|AHM93009.1|NADPH-cytochrome P450 reductase [*Sogatella furcifera*]	1332	0	100%
Putative acetylcholinesterase 1	KU764439	1585	483	partial	gb|ADR73026.1|putative acetylcholinesterase 1 [*Laodelphax striatella*]	832	0	83%
Angiotensin converting enzyme	KU764440	3803	664	Complete	gb|AGC79111.1|angiotensin converting enzyme [*Nilaparvata lugens*]	1234	0	93%
Carboxylesterase	KU764441	4793	570	Complete	gb|AFN66415.1|carboxylesterase, partial [*Laodelphax striatella*]	781	0	93%
**Immune related**								
Prophenoloxidase activating factor 2	KU764426	2250	451	Complete	gb|AID60320.1|prophenoloxidase activating factor 2, partial [*Nilaparvata lugens*]	560	0	86%
Serine protease snake-2	KU764427	1514	402	Complete	gb|AGK40920.1|serine protease snake-2 [*Nilaparvata lugens*]	701	0	90%
Serine protease snake-4	KU764428	2010	256	partial	gb|AGK40922.1|serine protease snake-4 [*Nilaparvata lugens*]	437	4e-148	79%
Serine protease snake-6	KU764429	1259	365	partial	gb|AGK40924.1| serine protease snake-6 [*Nilaparvata lugens*]	619	0	79%
**General digestion**								
Serine protease-11	KU764430	1339	401	partial	gb|AID60331.1|serine protease-11 [*Nilaparvata lugens*]	815	0	94%
**Other proteins**								
Uridine phosphorylase 1 isoform X2	KU764443	1569	350	Complete	ref|XP_014296528.1|PREDICTED: uridine phosphorylase 1 isoform X2 [*Microplitis demolitor*]	473	3e-163	76%
Putative phosphorylase b kinase regulatory subunit beta	KU764444	3507	1085	Complete	gb|KDR15354.1|putative phosphorylase b kinase regulatory subunit beta [*Zootermopsis nevadensis*]	1689	0	77%
Prostatic acid phosphatase-like isoform X1	KU764445	1603	438	Complete	ref|XP_012274118.1|PREDICTED: prostatic acid phosphatase-like isoform X1 [*Orussus abietinus*]	332	3e-106	48%
Prostatic acid phosphatase	KU764446	2641	429	partial	ref|XP_005175110.1|PREDICTED: prostatic acid phosphatase [*Musca domestica*]	372	3e-120	50%
Rac-GTP binding protein	KU764447	2496	641	Complete	dbj|BAN20830.1|rac-GTP binding protein [*Riptortus pedestris*]	856	0	66%
Calcium/calmodulin-dependent protein kinase 2 isoform X2	KU764448	5414	458	partial	ref|XP_014274099.1|PREDICTED: calcium/calmodulin-dependent protein kinase kinase 2 isoform X2 [*Halyomorpha halys*]	538	0	70%
Calcium/calmodulin-dependent protein kinase type 1 isoform X3	KU764449	8796	364	partial	ref|XP_012259790.1|PREDICTED: calcium/calmodulin-dependent protein kinase type 1 isoform X3 [*Athalia rosae*]	624	0	88%

### Tissue-specific expression patterns

**Fig 3:**
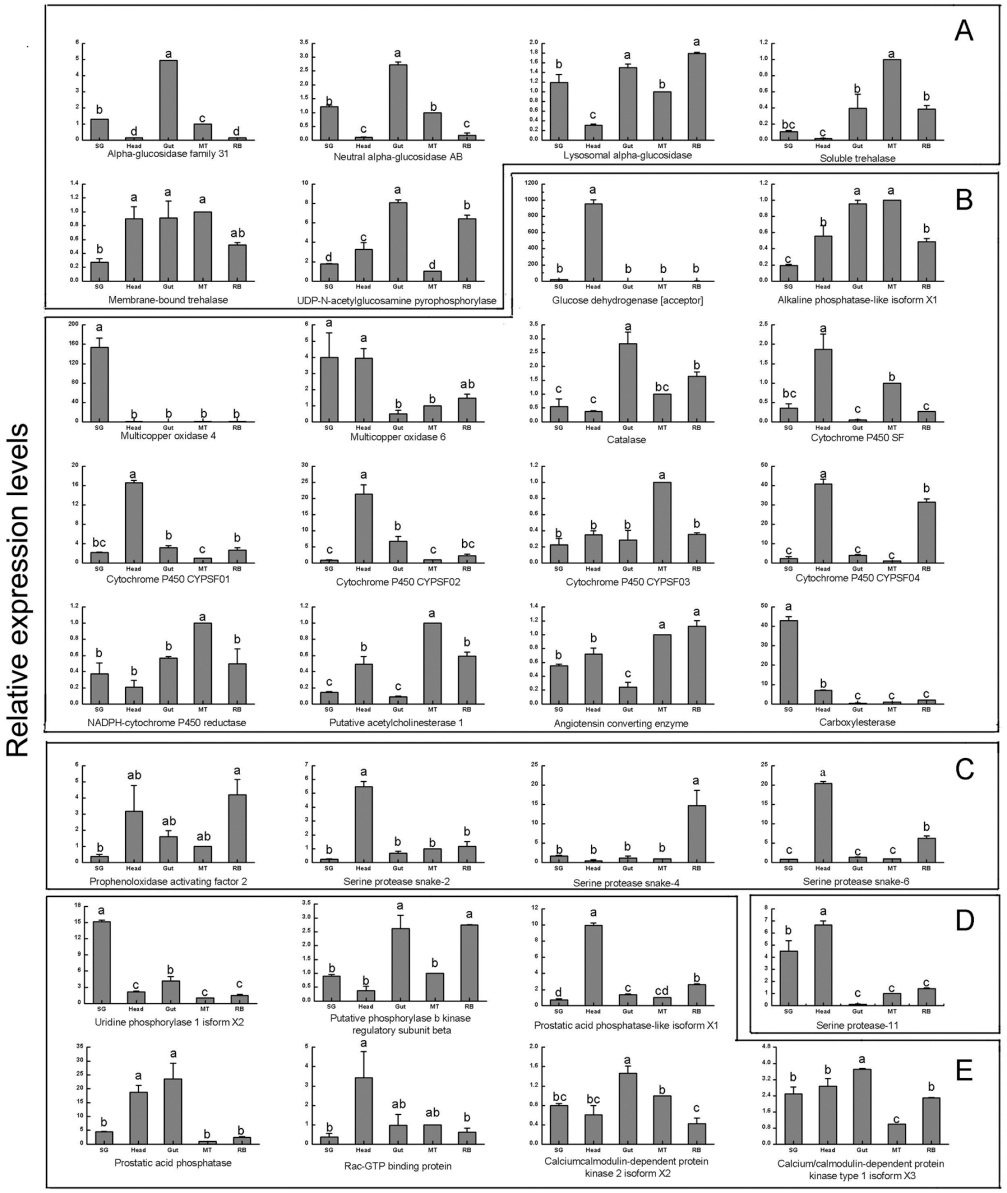
Transcript abundances of WBPH salivary protease genes in different tissues as measured by RT-qPCR. SG: salivary gland; Head: head without salivary gland; Gut: gut; MT: malpighian tubule; RB: remaining body (without salivary gland, head, gut and malpighian tubule). (A) Sugar metabolism; (B) Detoxification and inhibition of plant defenses; (C) Immune related; (D) General digestion; (E) Other proteins.

The expression of the 32 WBPH salivary protein genes in different tissues (SG, head, gut, MT, and RB) were determined by using real-time qPCR. With the expression level in MT for reference, all of the salivary protein genes were expressed in all tissues, but their expression levels had significant differences (Fig 3).

In this study, we identified six genes involved in sugar metabolism. The alpha-glucosidase family 31 and neutral alpha-glucosidase AB were expressed higher in gut than in other tissues. The transcript of lysosomal alpha-glucosidase was higher in the gut and RB than in other tissues. The expression level of soluble trehalase was the highest in MT among the different tissues, and membrane-bound trehalase had the same expression level in the different tissues except in SG which was the lowest. The transcript of UDP-*N*-acetylglucosamine pyrophosphorylase was higher in gut than in other tissues.

We investigated 14 salivary protein genes involved in detoxification and inhibition of plant defences. Glucose dehydrogenase [acceptor] had significantly 950.0 times higher expression levels in the head than in other tissues, while its expression levels in other tissues had no significant difference. The expression level of alkaline phosphatase-like isoform X1 was higher both in the gut and MT than in the head, RB and SG. Multicopper oxidase 4 expressed mainly in SG, and the expression level of multicopper oxidase 6 was about 4.0 times higher in SG and head than in MT. The transcript of catalase was the highest in the gut, while the expression levels of cytochrome P450 SF, cytochrome P450 CYPSF01, cytochrome P450 CYPSF02 and cytochrome P450 CYPSF04 was about 1.8, 16.5, 22.0 and 41.0 times higher in head than in MT. The expression levels of cytochrome P450 CYPSF03, NADPH-cytochrome P450 reductase and putative acetylcholinesterase 1 were the highest in MT among the different tissues. Carboxylesterase showed an expression level of about 43.0 times higher in SG than in MT.

In this study, four genes involved in immune related and one gene involved in general digestion were identified. Prophenoloxidase activating factor 2 had a lower expression levels in SG than in other four tissues, while serine protease snake-2 had significantly 5.0 times higher expression levels in the head than in other tissues, while its expression levels in other tissues had no significant difference. Serine protease snake-4 was expressed higher in RB compared with in other tissues. The expression levels of serine protease snake-6 and serine protease-11 were about 20.0 and 6.5 times higher in head than in MT. however angiotensin converting enzyme was expressed the lowest in the gut among the five tissues.

Seven other proteins involved in signal pathway and metabolism were investigated in this study. Uridine phosphorylase 1 isoform X2 showed an expression level of about 15.0 times higher in SG than in MT. The transcripts of putative phosphorylase b kinase regulatory subunit beta were higher in gut and RB than in other tissues. The expression levels of prostatic acid phosphatase-like isoform X1 and prostatic acid phosphatase were about 9.5 and 18.0 times higher in head than in MT, while the latter was also expressed higher in gut than in SG, RB and MT. The expression level of Rac-GTP binding protein had no difference in head, gut and MT. The expression level of calcium/calmodulin-dependent protein kinase 2 isoform X2 was the highest in gut and the lowest in RB, but there was no difference among in SG, head and MT. Calcium/calmodulin-dependent protein kinase type 1 isoform X3 showed the highest expression level in gut and the lowest in MT compared with other tissues.

### Development and sex-specific expression patterns

**Fig 4:**
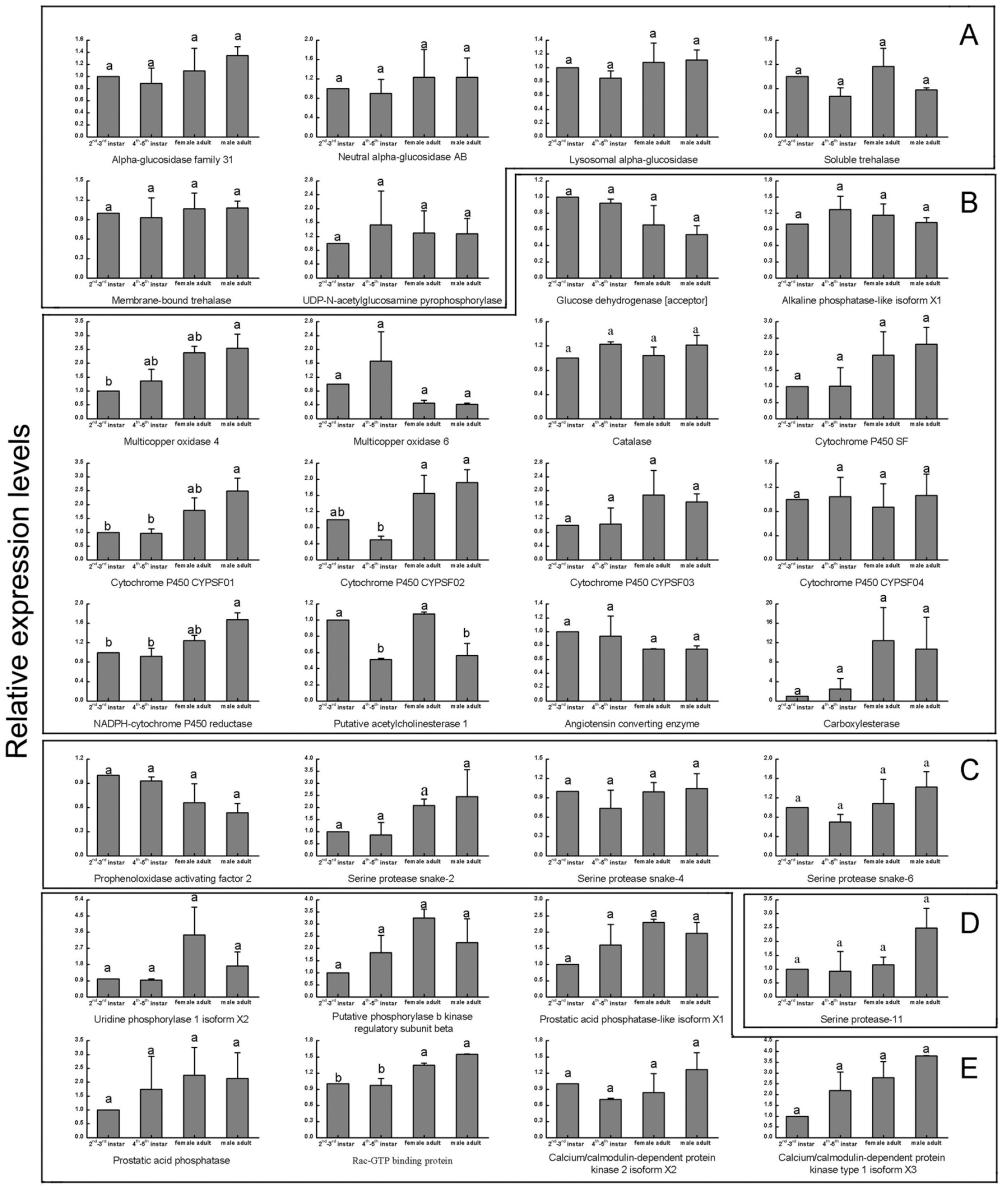
Developmental stage- and sex-specific expression of salivary protease genes in WBPH salivary gland by RT-qPCR. Total RNA was extracted from the salivary gland of 2^nd^-3^rd^ instar nymph, 4^th^-5^th^ instar nymph, female and male adult. (A) Sugar metabolism; (B) Detoxification and inhibition of plant defenses; (C) Immune related; (D) General digestion; (E) Other proteins.

The transcript abundances of 32 salivary protein genes in SG of WBPH 2^nd^-3^rd^ instar nymph, 4^th^-5^th^ instar nymph, female and male adult were investigated by real-time qPCR. The significantly differentiation of development and sex-specific expressions of 32 salivary protein genes were shown in Fig 4. With the expression level in SG of 2^nd^-3^rd^ instar nymph for reference, most of the salivary proteins had similar expression levels in SG of developmental stages and sexes, except multicopper oxidase 4, cytochrome P450 CYPSF01, cytochrome P450 CYPSF02, NADPH-cytochrome P450 reductase, putative acetylcholinesterase 1, and Rac-GTP binding protein. The expression level of multicopper oxidase 4 was increased from the 2^nd^-3^rd^ nymph to adult stages. The relatively transcript levels of cytochrome P450 CYPSF01 and NADPH-cytochrome P450 reductase were higher in adult than in instar. Cytochrome P450 CYPSF02 showed a lower expression level in 4^th^-5^th^ instar than that in other development stages. The putative acetylcholinesterase 1 was expressed higher in the stages of the 2^nd^-3^rd^ instar and female adult than those in the stages of the 4^th^-5^th^ instar and male adult. Rac-GTP binding protein had a lower expression level at the stages of 2^nd^-3^rd^ and 4^th^-5^th^ instar than that in adult stage. Except putative acetylcholinesterase 1, the expression levels of other 31 enzymes had no difference between female and male adult.

## Discussion

Salivary enzymes of Hemiptera played a key role between the insect and plant. Despite there are some studies, but more thorough system of related gene research is less. In this study, we obtained 51,407 unigenes with mean lengths of 647 bp, which are a major genomic resource for investigating the salivary glands of the WBPH. The total number of unigenes obtained was much higher than the numbers of salivary gland unigenes identified in *N*. *lugens* (43,312 unigenes) [2] and in *B*. *tabaci* (13,615 unigenes) [5], which reflects the sequencing depth and coverage. In this study, 32 salivary protein genes were studied using RT-PCR and real-time qPCR in order to comprehensive evaluation of the WBPH sialotranscripome.

### Tissue-specific expression patterns

Alpha-glucosidase family 31, neutral alpha-glucosidase AB, and lysosomal alpha-glucosidase were the enzyme types typically involved in sugar metabolism and potentially in detoxification and plant defence suppression [13]. The three glucosidases were all found in the WBPH sialotranscriptome. The gene transcripts of alpha-glucosidase family 31 and neutral alpha-glucosidase AB were detected at the highest level in the gut followed by SG and MT. Consistent with our results, α-glucosidase was active in the midgut and salivary, but the expression levels of α-glucosidase was found significantly higher in the midgut than in salivary gland of *Brachynema germari* [25]. Lysosomal alpha-glucosidase-like had a high expression levels both in gut and RB, followed by SG and MT, but was hardly detected in the head. It was suggested that these enzymes may have a role of digestion and detoxification in gut.

Trehalases play a key role in various trehalose-associated physiological processes in insects, including fight metabolism [26], chitin synthesis during molting [27, 28], and development [29]. There were two types of trehalases (soluble trehalases and membrane-bound trehalase) in insect, but they may have different roles in the physiological process [28, 30]. In this study, we found the transcripts of soluble trehalases were the highest in MT and the lowest in the head. The expression level of membrane-bound trehalase was lower in SG than in other tissues. The transcript of soluble trehalases in different tissues of *Aphis glycines* was higher in the gut compared to in the integument, fat body and embryo [31], the expressions of soluble trehalases were similar with the findings in *Spodoptera frugiperda* and *B*. *mori* [28, 32].

UDP-*N*-acetylglucosamine pyrophosphorylase (UAP) was an enzyme of chitin biosynthetic pathway which began with trehalose [33]. UAP for the development of *L*. *migratoria* showed that both *LmUAP1* and *LmUAP2* were widely expressed in all the major tissues besides chitin-containing tissues [34], especially *LmUAP1* had the highest expression levels in the integument and the highest levels of *LmUAP2* was found in the fat bodies of the 5^th^ instar. In this study, UAP was identified to have the highest transcripts in the gut of WBPH, and followed by RB, head, SG and MT. Uridine phosphorylase 1 isoform X2 showed the highest expression level in SG among the five tissues in WBPH.

Glucose dehydrogenase was also found in the saliva of *D*. *noxia* [35], *Myzus persicae* [36], and *A*. *pisum* [37], and it was also identified to have higher expression level in the head than in other tissues (thorax and abdomen) of *Apis mellifera* [38].

Alkaline phosphatase played a key role in several biological processes and responded to stress, pathogenesis, or infection [39–44]. Alkaline phosphatase-like isoform X1 was identified in the WBPH. The transcripts of the enzyme were higher in the gut and MT followed by in the head, RB and SG. The expression of alkaline phosphatase in the silkworm was confined mainly in the gut tissue [42]. Two forms (soluble and membrane-bound) of alkaline phosphatase (sALP and mALP) was identified in *N*. *lugens* [17], however alkaline phosphatase-like isoform X1 in WBPH was difficult to judge belong to sALP or mALP, because both sALP and mALP show approximately the same degree of sequences identity (43.74%-45.97%) to the alkaline phosphatase-like isoform X1. The functions of alkaline phosphatase-like isoform X1 in WBPH will be investigated in future.

There were multiple enzymes of laccases in the multicopper oxidase (MCO) family. The largest subgroup of MCOs of laccases is present in plants, fungi, bacteria and insects [14]. In this study, qPCR revealed MCO4 was expressed primarily in SG, which is similar to that of the MCO4 in *N*. *lugens* [14]. MCO6 was expressed higher in SG, head and RB among WBPH tissues, the result was similar to that in *N*. *lugens* [14]. When *N*. *lugens* was injected of MCO6 dsRNA, it led to shedding failure of old cuticles, wing deformities and a high mortality, similar to MCO6 in *T*. *castaneum* and *Anopheles gambiae*, these was a strong evidence of MCO6 might play a role during molting [14, 45]. But the functions of MCO6 in WBPH need further to be clarified.

Catalase is the main enzyme that decomposes hydrogen peroxide (H_2_O_2_) at high concentrations. H_2_O_2_ is a part of the free radicals system that mediates important physiological roles including signalling and defence. In this study, we found that the transcript of catalase had the maximum expression level in the gut and MT followed by RB, SG and head. Catalase of *N*. *lugens* had the higher activity in the gut than salivary gland and whole body extracts, and the activity of catalase was higher in the salivary gland of BPH fed on a hopper-resistant rice variety than a susceptible rice variety, which revealed that the catalase had a most obvious role of direct toxicity from H_2_O_2_ [46].

Cytochrome P450s (CYRs) might be the major detoxification enzymes when the insect adapted to its chemical environment [47]. In this study, five sequences coding for CYRs were identified, and most of the transcripts were found to be expressed in the head of adult WBPH except CYPSF03 which were expressed highly in MT. The expression levels of 64 CYPs were identified in *Dendroctonus armandi*, suggesting that most of the CYPs were expressed in the larvae, pupae and adult [47]. The widely different expression patterns of CYPs in WBPH suggested that CYPs might have multiple functions in the different tissues. NADPH–cytochrome P450 reductase (CPR) (EC 1.6.2.4) is an essential enzyme which may play a specific function in P450-mediated detoxification pathways and other physiological processes in insects [48]. The transcripts of NADPH-cytochrome P450 reductase in WBPH were most abundant in MT in adult and were the lowest in SG of 2^nd^-3^rd^ instar nymphs. While in *N*. *lugens*, the expression levels of NADPH-cytochrome P450 reductase (*NlCPR*) were the highest in the abdomen in adults and in 1^st^ instar nymphs [49].

Acetylcholinesterase (AChE, EC 3.1.1.7) was a key enzyme that regulated the level of the neurotransmitter acetylcholine and terminated nerve impulses in the nervous system of insects [50]. The transcripts of putative acetylcholinesterase 1 in WBPH were most abundant in MT and the lowest in SG and gut compared with other tissues. AChE1 was identified to express abundantly in most tissues of *Chilo suppressalis* [51]. The AChE1 was expressed higher than AChE2 in different tissues of *N*. *lugens*, suggesting that it may be the main target of organophosphorus insecticides [52]. Insect angiotensin converting enzyme (ACE) is a zinc metallopeptidase and can inactivate a variety of small to medium size peptide hormones by cleavage of C-terminal dipeptides and dipeptideamides [16]. ACE has been characterised in many insects such as *Bombyz mori*, *D*. *melanogaster*, *Locusta migratoria*, *Musca domestica*, *Spodoptera littoralis* and *N*. *lugens* [53–58]. High expression level of ACE was found in RB and MT followed by SG, head and gut of WBPH. The transcripts of ACE were highly expressed in the hemolymph and reproductive tissues of both sexes in the adult *M*. *sexta* than in the brain, gut and fat body [16].

Carboxylesterase (CarE) was a ubiquitous enzyme of many functions in animals, plants and microorganism [59, 60]. It had crucial roles in xenobiotic detoxification, pheromone degradation, neurogenesis and developmental regulations [61]. It was verified that the expression levels of the enzymes were high in larvae maxilla of *B*. *mori*, suggesting that they may degrade plant volatiles or other xenobiotics [61]. We detected the carboxylesterase gene had a significant high expression level in SG of WBPH. The expression pattern suggested that it may play a role in detoxifying plant allelochemicals or degrading the plant-cell in the interaction of insect and plant.

Serine proteases play very important roles in the innate immune responses of invertebrate animals. These proteases are mainly involved in the processes of melanin formation and hemolymph coagulation against the infections of foreign pathogens [15]. A family of serine proteases has been discovered in invertebrates, and shown to include embryonic development and immune responses of diverse biological processes [62]. The serine protease snake 2 and 4 were also reported in *N*. *lugens* which have the similar expression patterns [15]. These serine protease genes had high expression levels in head, except snake 4 which was higher in RB than in the other tissues. The transcripts of prophenoloxidase activating factor 2 had no difference among these tissues except in SG which was lower. Prophenoloxidase activating factor 2 that had catalytic function is one of clip domain family of serine proteases [63, 64], Clip-domain serine proteases (CLIPs) played an important role in mediating innate immunity, called proPO activation cascade, embryonic development and hemolymph clotting in arthropods [65]. These results suggested that the serine protease genes might have functions in the different tissues.

Four phosphatases of uridine phosphorylase 1 isoform X2, putative phosphorylase b kinase regulatory subunit beta, prostatic acid phosphatase-like isoform X1 and prostatic acid phosphatase were identified in WBPH. Phosphorylase b kinase (PhK) controls glycogen degradation via phosphorylation of glycogen phosphorylase b (GPb) [66]. The transcripts of putative phosphorylase b kinase regulatory subunit beta were higher both in gut and RB compared with three other tissues in WBPH. The transcripts of prostatic acid phosphatase-like isoform X1 and prostatic acid phosphatase were both higher in the head than in other tissues, while prostatic acid phosphatase was also expressed highly in the gut.

The Ras superfamily of GTP-binding proteins was subdivided into Ras, Rho, Ran, Arf and Rab proteins [67], and it had a variety role to regulate the cellular activities. The Rac proteins belonged to Rho family, which played a role in the processes of assembling the actin cytoskeleton and the members of phagocyte NADPH oxidase [68]. In WBPH, Rac-GTP binding protein showed higher expression level in the head, gut and MT than in SG and RB.

Calcium/calmodulin-dependent protein kinase 2 (CaMKII) is an essential signalling kinase involved in neuronal plasticity in insects [69], different calcium/calmodulin-dependent protein kinase 2 isoforms have been cloned in *D*. *melanogaster* [70], *Apis florae* [71], *Bombus terrestris* [72], *M*. *sexta* [73, 74] and *Periplaneta americana* [69]. The transcripts of calcium/calmodulin-dependent protein kinase 2 isoform X2 in WBPH were detected in all tissues, and the expression levels of which were higher in the gut, SG and head compared with others tissues. The calcium/calmodulin-dependent protein kinase type 1 isoform X3 showed similar expression pattern.

### Development and sex-specific expression patterns

In this study, we obtained WBPH development and sex-specific expression profile data of 32 salivary protein genes of SG for 2^nd^-3^rd^ instar nymphs, 4^th^-5^th^ instar nymphs, female and male adults. 26 of 32 salivary protein genes (except MCO4, NADPH-cytochrome P450 reductase, cytochrome P450 CYPSF01, cytochrome P450 CYPSF02, putative acetylcholinesterase 1 and Rac-GTP binding protein) had similar expression levels in SG of developmental stages and sexes, suggesting that they were like to have multiple functions in SG of all developmental stages and sexes. Except cytochrome P450 CYPSF02 and putative acetylcholinesterase 1, the expression levels of other four salivary protein genes were higher in adult stages than in nymph stages, while the sexes had no difference between them, suggesting that these enzymes may play a more important role in SG of adult. The transcripts expression levels of cytochrome P450 CYPSF02 were lower in 4^th^-5^th^ instar than that in other development stages. Putative acetylcholinesterase 1 was expressed higher in SG of 2^nd^-3^rd^ instar and female adult than in 4^th^-5^th^ instar and male adult. 31 of 32 salivary protein genes (except putative acetylcholinesterase 1) showed no difference of expression levels between the female and male adult, suggesting that these enzymes had similar expression levels in SG of sexes. The detailed functions and relationships between the salivary protein genes in SG of development stages and sexes were needed to be further clarified.

## Supporting Information

S1 Table Gene specific primers used for molecular cloning of WBPH salivary protein genes. (XLSX)

S2 Table Primers used in real-time qPCR for determination expression level of WBPH salivary protein genes. (XLSX)

## References

[1] Will T, Furch ACU, Zimmermann MR. How phloem–feeding insects face the challenge of phloem–located defenses. Front Plant Sci. 2013; 4: 336 10.3389/fpls.2013.00336 24009620 PMC3756233

[2] Ji R, Yu HX, Fu Q, Chen HD, Ye WF, Li SH, et al Comparative transcriptome analysis of salivary glands of two populations of rice brown planthopper, *Nilaparvata lugens*, that differ in virulence. PloS One. 2013; 8(11): e79612 10.1371/journal.pone.0079612 24244529 PMC3828371

[3] Miles PW. Aphid saliva. Biol Rev. 1999; 74(1): 41–85.

[4] Sharma A, Khan AN, Subrahmanyam S, Raman A, Taylor GS, Fletcher MJ. Salivary proteins of plant–feeding hemipteroids–implication in phytophagy. Bull Entomol Res. 2014; 104(2): 117–136. 10.1017/S0007485313000618 24280006

[5] Su YL, Li JM, Li M, Luan JB, Ye XD, Wang XW, et al Transcriptomic analysis of the salivary glands of an invasive whitefly. PLoS One. 2012; 7(6): e39303 10.1371/journal.pone.0039303 22745728 PMC3379992

[6] Blanc S, Uzest M, Drucker M. New research horizons in vector–transmission of plant viruses. Curr Opin Microbiol. 2011; 14(4): 483–491. 10.1016/j.mib.2011.07.008 21788152

[7] Fujita D, Kohli A, Horgan FG. Rice resistance to planthoppers and leafhoppers. Crit Rev Plant Sci. 2013; 32(3): 162–191.

[8] Matsukura K, Towata T, Sakai J, Onuki M, Okuda M, Matsumura M. Dynamics of southern rice black–streaked dwarf virus in rice and implication for virus acquisition. Phytopathology. 2013; 103(5): 509–512. 10.1094/PHYTO-10-12-0261-R 23301813

[9] Otuka A. Migration of rice planthoppers and their vectored re–emerging and novel rice viruses in East Asia. Front Microbiol. 2013; 4: 309 10.3389/fmicb.2013.00309 24312081 PMC3836001

[10] Zhao W, Lu LX, Yang PC, Cui N, Kang L, Cui F. Organ–specific transcriptome response of the small brown planthopper toward rice stripe virus. Insect Biochem Mol Biol. 2016; 70: 60–72. 10.1016/j.ibmb.2015.11.009 26678499

[11] Matsumoto Y, Suetsugu Y, Nakamura M, Hattori M. Transcriptome analysis of the salivary glands of *Nephotettix cincticeps* (Uhler). J Insect Physiol. 2014; 71: 170–176. 10.1016/j.jinsphys.2014.10.010 25450428

[12] DeLay B, Mamidala P, Wijeratne A, Wijeratne S, Mittapalli O, Wang J, et al Transcriptome analysis of the salivary glands of potato leafhopper, *Empoasca fabae*. J Insect Physiol. 2012; 58(12): 1626–1634. 10.1016/j.jinsphys.2012.10.002 23063500

[13] Nicholson SJ, Hartson SD, Puterka GJ. Proteomic analysis of secreted saliva from Russian wheat aphid (Diuraphis noxia Kurd.) biotypes that differ in virulence to wheat. J Proteomics. 2012; 75(7): 2252–2268. 10.1016/j.jprot.2012.01.031 22348819

[14] Ye YX, Pan PL, Kang D, Lu JB, Zhang CX. The multicopper oxidase gene family in the brown planthopper, *Nilaparvata lugens*. Insect Biochem Mol Biol. 2015; 63: 124–132. 10.1016/j.ibmb.2015.06.010 26107750

[15] Bao YY, Qin X, Yu B, Chen LB, Wang ZC, Zhang CX. Genomic insights into the serine protease gene family and expression profile analysis in the planthopper, *Nilaparvata lugens*. BMC genomics. 2014; 15: 507 10.1186/1471-2164-15-507 24952583 PMC4085338

[16] Isaac RE, Lamango NS, Ekbote U, Taylor CA, Hurst D, Weaver RJ, et al Angiotensin–converting enzyme as a target for the development of novel insect growth regulators. Peptides. 2007; 28(1): 153–162. 17157962 10.1016/j.peptides.2006.08.029

[17] Wang ZX, Liu SH, Yang BJ, Liu ZW. Characterization of soluble and membrane–bound alkaline phosphatase in *Nilaparvata lugens* and their potential relation to development and insecticide resistance. Arch Insect Biochem Physiol. 2011; 78(1): 30–45. 10.1002/arch.20437 21769927

[18] Wang J, He WB, Su YL, Bing XL, Liu SS. Molecular characterization of soluble and membrane–bound trehalases of the whitefly, *Bemisia tabaci*. Arch Insect Biochem Physiol. 2014; 85(4): 216–233. 10.1002/arch.21155 24610752

[19] Grabherr MG, Haas BJ, Yassour M, Levin JZ, Thompson DA, Amit I, et al Full–length transcriptome assembly from RNA–Seq data without a reference genome. Nat Biotechnol. 2011; 29(7): 644–652. 10.1038/nbt.1883 21572440 PMC3571712

[20] Conesa A, Götz S, García-Gómez JM, Terol J, Talón M, Robles M. Blast2GO: a universal tool for annotation, visualization and analysis in functional genomics research. Bioinformatics. 2005; 21(18): 3674–3676. 16081474 10.1093/bioinformatics/bti610

[21] An XK, Hou ML, Liu YD. Reference gene selection and evaluation for gene expression studies using qRT–PCR in the white–backed planthopper, *Sogatella furcifera* (Hemiptera: Delphacidae). J Econ Entomol. 2015; 109(2):879–886. 10.1093/jee/tov333 26612891

[22] Livak KJ, Schmittgen TD. Analysis of relative gene expression data using real–time quantitative PCR and the 2^-ΔΔCT^ Method. Methods. 2001; 25(4): 402–408. 11846609 10.1006/meth.2001.1262

[23] Xue J, Bao YY, Li BL, Cheng YB, Peng ZY, Liu H, et al Transcriptome analysis of the brown planthopper *Nilaparvata lugens*. PLoS One. 2010; 5(12): e14233 10.1371/journal.pone.0014233 21151909 PMC2997790

[24] Xu Y, Zhou W, Zhou YJ, Wu JX, Zhou XP. Transcriptome and comparative gene expression analysis of *Sogatella furcifera* (Horváth) in response to southern rice black–streaked dwarf virus. PLoS One. 2012; 7(4): e36238 10.1371/journal.pone.0036238 22558400 PMC3338671

[25] Ramzi S, Hosseininaveh V. Biochemical characterization of digestive α–amylase, α–glucosidase and β–glucosidase in pistachio green stink bug, *Brachynema germari* Kolenati (Hemiptera: Pentatomidae). J Asia Pac Entomol. 2010; 13(3): 215–219.

[26] Clegg JS, Evans DR. Blood trehalose and flight metabolism in the blowfly. Science. 1961; 134(3471): 54–55. 13694011 10.1126/science.134.3471.54

[27] Tatun N, Singtripop T, Sakurai S. Dual control of midgut trehalase activity by 20–hydroxyecdysone and an inhibitory factor in the bamboo borer *Omphisa fuscidentalis* Hampson. J Insect Physiol. 2008; 54(2): 351–357. 18023454 10.1016/j.jinsphys.2007.10.006

[28] Chen J, Tang B, Chen HX, Yao Q, Huang XF, Chen J, et al Different functions of the insect soluble and membrane–bound trehalase genes in chitin biosynthesis revealed by RNA interference. PloS One. 2010; 5(4): e10133 10.1371/journal.pone.0010133 20405036 PMC2853572

[29] Tatun N, Singtripop T, Tungjitwitayakul J, Sakurai S. Regulation of soluble and membrane–bound trehalase activity and expression of the enzyme in the larval midgut of the bamboo borer *Omphisa fuscidentalis*. Insect Biochem Mol Biol. 2008; 38(8): 788–795. 10.1016/j.ibmb.2008.05.003 18625402

[30] Ge LQ, Zhao KF, Huang LJ, Wu JC. The effects of triazophos on the trehalose content, trehalase activity and their gene expression in the brown planthopper *Nilaparvata lugens* (Stål) (Hemiptera: Delphacidae). Pestic Biochem Physiol. 2011; 100(2): 172–181. 21760647 10.1016/j.pestbp.2011.03.007 PMC3102831

[31] Bansal R, Mian MAR, Mittapalli O, Michel AP. Molecular characterization and expression analysis of soluble trehalase gene in *Aphis glycines*, a migratory pest of soybean. Bull Entomol Res. 2013; 103(3): 286–295. 10.1017/S0007485312000697 23445549

[32] Silva MCP, Ribeiro AF, Terra WR, Ferreira C. Sequencing of *Spodoptera frugiperda* midgut trehalases and demonstration of secretion of soluble trehalase by midgut columnar cells. Insect Mol Biol. 2009; 18(6): 769–784. 10.1111/j.1365-2583.2009.00920.x 19843188

[33] Merzendorfer H, Zimoch L. Chitin metabolism in insects: structure, function and regulation of chitin synthases and chitinases. J Exp Biol. 2003; 206(24): 4393–4412. 14610026 10.1242/jeb.00709

[34] Liu XJ, Li F, Li DQ, Ma EB, Zhang WQ, Zhu KY, et al Molecular and functional analysis of UDP–*N*–acetylglucosamine pyrophosphorylases from the migratory locust, *Locusta migratoria*. PloS One. 2013; 8(8): e71970 10.1371/journal.pone.0071970 23977188 PMC3747057

[35] Cooper WR, Dillwith JW, Puterka GJ. Salivary proteins of Russian wheat aphid (Hemiptera: Aphididae). Envir Entomol. 2010; 39(1): 223–231. 10.1603/EN09079 20146860

[36] Harmel N, Létocart E, Cherqui A, Giordanengo P, Mazzucchelli G, Guillonneau F, et al Identification of aphid salivary proteins: a proteomic investigation of *Myzus persicae*. Insect Mol Biol. 2008; 17(2): 165–174. 10.1111/j.1365-2583.2008.00790.x 18353105

[37] Carolan JC, Fitzroy CIJ, Ashton PD, Douglas AE, Wilkinson TL. The secreted salivary proteome of the pea aphid *Acyrthosiphon pisum* characterised by mass spectrometry. Proteomics. 2009; 9(9): 2457–2467. 10.1002/pmic.200800692 19402045

[38] Kucharski R, Maleszka R. Evaluation of differential gene expression during behavioral development in the honeybee using microarrays and northern blots. Genome Biol. 2002; 3(2): 1–0007. 10.1186/gb-2002-3-2-research0007 PMC65684 11864369

[39] Kucera M, Weiser J. Alkaline phosphatase in the last larval instar of *Barathra brassicae* (Lepidoptera) infected by Nosema plodiae. Acta Entomol Bohemoslov. 1974; 71: 3127–3135.

[40] Sujak P, Ziemnicki K, Ziemnicka J, Lipa JJ, Obuchowicz L. Acid and alkaline phosphatase activity in the fat body and midgut of the beet armyworm, *Spodoptera exigua* (Lepidoptera: Noctuidae), infected with nuclear polyhedrosis virus. J Invertebr Pathol. 1978; 31(1): 4–9.

[41] Chang WS, Zachow KR, Bentley D. Expression of epithelial alkaline phosphatase in segmentally iterated bands during grasshopper limb morphogenesis. Development. 1993; 118(2): 651–663. 8223284 10.1242/dev.118.2.651

[42] Eguchi M. Alkaline phosphatase isozymes in insects and comparison with mammalian enzyme. Comp Biochem Phys B. 1995; 111(2): 151–162. 10.1016/0305-0491(94)00248-s 7599983

[43] Sukhanova MJ, Grenback LG, Gruntenko NE, Khlebodarova TM, Rauschenbach IY. Alkaline phosphatase in *Drosophila* under heat stress. J Insect Physiol. 1996; 42(2): 161–165.

[44] Miao YG. Studies on the activity of the alkaline phosphatase in the midgut of infected silkworm, *Bombyx mori* L. J Appl Ent. 2002; 126(2–3): 138–142.

[45] Peng Z, Green PG, Arakane Y, Kanost MR, Gorman MJ. A multicopper oxidase–related protein is essential for insect viability, longevity and ovary development. PloS One. 2014; 9(10): e111344 10.1371/journal.pone.0111344 25330116 PMC4203857

[46] Petrova A, Smith CM. Immunodetection of a brown planthopper *(Nilaparvata lugens* Stål) salivary catalase–like protein into tissues of rice, *Oryza sativa*. Insect Mol Biol. 2014; 23(1): 13–25. 10.1111/imb.12058 24164290

[47] Dai LL, Ma MY, Wang CY, Shi Q, Zhang RR, Chen H. Cytochrome P450s from the Chinese white pine beetle, *Dendroctonus armandi* (Curculionidae: Scolytinae): Expression profiles of different stages and responses to host allelochemicals. Insect Biochem Mol Biol. 2015; 65: 35–46. 10.1016/j.ibmb.2015.08.004 26319543

[48] Paine MJI, Scrutton NS, Munro AW, Gutierrez A, Roberts GCK, Wolf CR. Electron transfer partners of cytochrome P450 Cytochrome P450: Springer US; 2005 pp. 115–148.

[49] Liu S, Liang QM, Zhou WW, Jiang YD, Zhu QZ, Yu H, et al RNA interference of NADPH–cytochrome P450 reductase of the rice brown planthopper, *Nilaparvata lugens*, increases susceptibility to insecticides. Pest Manag Sci. 2015; 71(1): 32–39. 10.1002/ps.3760 24515640

[50] Toutant JP. Insect acetylcholinesterase: catalytic properties, tissue distribution and molecular forms. Prog Neurobiol. 1989; 32(5): 423–446. 2660188 10.1016/0301-0082(89)90031-2

[51] Jiang XJ, Qu MJ, Denholm I, Fang JC, Jiang WH, Han ZJ. Mutation in acetylcholinesterase1 associated with triazophos resistance in rice stem borer, *Chilo suppressalis* (Lepidoptera: Pyralidae). Biochem Biophys Res Commun. 2009; 378(2): 269–272. 10.1016/j.bbrc.2008.11.046 19028456

[52] Li BL, Chen W, Liu L, Zhang XC, Bao YY, Cheng JA, et al Molecular characterization of two acetylcholinesterase genes from the brown planthopper, *Nilaparvata lugens* (Hemiptera: Delphacidae). Pestic Biochem Physiol. 2012; 102(3): 198–203.

[53] Quan GX, Mita K, Okano K, Shimada T, Ugajin N, Xia Z, et al Isolation and expression of the ecdysteroid–inducible angiotensin–converting enzyme–related gene in wing discs of *Bombyx mori*. Insect Biochem Mol Biol. 2001; 31(1): 97–103. 11102839 10.1016/s0965-1748(00)00112-0

[54] Siviter RJ, Taylor CAM, Cottam DM, Denton A, Dani MP, Milner MJ, et al Ance, a Drosophila angiotensin–converting enzyme homologue, is expressed in imaginal cells during metamorphosis and is regulated by the steroid, 20–hydroxyecdysone. Biochem J. 2002; 367(1): 187–193. 12093364 10.1042/BJ20020567 PMC1222869

[55] Isaac RE, Schoofs L, Williams AT, Veelaert D, Sajid M, Corvol P, et al A novel peptide–processing activity of insect peptidyl–dipeptidase A (angiotensin I–converting enzyme): the hydrolysis of lysyl–arginine and arginyl–arginine from the C–terminus of an insect prohormone peptide. Biochem J. 1998; 330(1): 61–65. 9461491 10.1042/bj3300061 PMC1219108

[56] Lamango NS, Nachman RJ, Hayes TK, Strey A, Isaac RE. Hydrolysis of insect neuropeptides by an angiotensin–converting enzyme from the housefly, *Musca domestica*. Peptides. 1997; 18(1): 47–52. 9114451 10.1016/s0196-9781(96)00232-x

[57] Lemeire E, Vanholme B, Leeuwen TV, Camp JV, Smagghe G. Angiotensin–converting enzyme in *Spodoptera littoralis*: molecular characterization, expression and activity profile during development. Insect Biochem Mol Biol. 2008; 38(2): 166–175. 10.1016/j.ibmb.2007.10.004 18207078

[58] Sun ZX, Zhai YF, Zhang JQ, Kang K, Cai JH, Fu Y, et al The genetic basis of population fecundity prediction across multiple field populations of *Nilaparvata lugens*. Mol Ecol. 2015; 24(4): 771–784. 10.1111/mec.13069 25581109

[59] Marshall SDG, Putterill JJ, Plummer KM, Newcomb RD. The carboxylesterase gene family from *Arabidopsis thaliana*. J Mol Evol. 2003; 57(5): 487–500. 14738307 10.1007/s00239-003-2492-8

[60] Bornscheuer UT. Microbial carboxyl esterases: classication, properties and application in biocatalysis. FEMS Microbiol Rev. 2002; 26(1): 73–81. 12007643 10.1111/j.1574-6976.2002.tb00599.x

[61] Yu QY, Lu C, Li WL, Xiang ZH, Zhang Z. Annotation and expression of carboxylesterases in the silkworm, *Bombyx mori*. BMC genomics. 2009; 10: 553 10.1186/1471-2164-10-553 19930670 PMC2784812

[62] Muta T, Oda T, Iwanaga S. Horseshoe crab coagulation factor B. A unique serine protease zymogen activated by cleavage of an Ile–Ile bond. J Biol Chem. 1993; 268(28): 21384–21388. 8407978

[63] Piao S, Kim D, Park JW, Lee BL, Ha NC. Overexpression and preliminary X–ray crystallographic analysis of prophenoloxidase activating factor II, a clip domain family of serine proteases. Biochim Biophys Acta. 2005; 1752(1): 103–106. 15953772 10.1016/j.bbapap.2005.05.008

[64] Piao S, Song YL, Kim JH, Park SY, Park JW, Lee BL, et al Crystal structure of a clip–domain serine protease and functional roles of the clip domains. EMBO J. 2015; 24(24): 4404–4414. 10.1038/sj.emboj.7600891 PMC1356332 16362048

[65] Bao YY, Qu LY, Zhao D, Chen LB, Jin HY, Xu Lm, et al The genome–and transcriptome–wide analysis of innate immunity in the brown planthopper, *Nilaparvata lugens*. BMC genomics. 2013; 14(1). 10.1186/1471-2164-14-160 PMC3616906 23497397

[66] Cohen P, Burchell A, Foulkes JG, Cohen PTW, Vanaman TC, Nairin AC. Identification of the Ca^2+^–dependent modulator protein as the fourth subunit of rabbit skeletal muscle phosphorylase kinase. FEBS Lett. 1978; 92(2): 287–293. 212300 10.1016/0014-5793(78)80772-8

[67] Gasper R, Sot B, Wittinghofer A. GTPase activity of Di–Ras proteins is stimulated by Rap1GAP proteins. Small GTPases. 2010; 1(3): 133–141. 21686267 10.4161/sgtp.1.3.14742 PMC3116609

[68] Xu X, Barry DC, Settleman J, Schwartz MA, Bokoch GM. Differing structural requirements for GTPase–activating protein responsiveness and NADPH oxidase activation by Rac. J Biol Chem. 1994; 269(38): 23569–23574. 8089125

[69] Taillebois E, Heuland E, Bourdin CM, Griveau A, Quinchard S, Tricoire–Leignel H, et al Ca^2+^/calmodulin–dependent protein kinase II in the cockroach *Periplaneta americana*: identification of five isoforms and their tissues distribution. Arch Insect Biochem Physiol. 2013; 83(3): 138–150. 10.1002/arch.21102 23740573

[70] Ohsako S, Nishida Y, Ryo H, Yamauchi T. Molecular characterization and expression of the Drosophila Ca^2+^/calmodulin–dependent protein kinase II gene. Identification of four forms of the enzyme generated from a single gene by alternative splicing. Journal Biol Chem. 1993; 268(3): 2052–2062. 8380587

[71] Kamikouchi A, Takeuchi H, Sawata M, Natori S, Kubo T. Concentrated expression of Ca^2+^/calmodulin–dependent protein kinase II and protein kinase C in the mushroom bodies of the brain of the honeybee *Apis mellifera* L. J Comp Neurol. 2000; 417(4): 501–510. 10701869 10.1002/(sici)1096-9861(20000221)417:4<501::aid-cne8>3.0.co;2-4

[72] Inagaki S, Kaku K, Dunlap DY, Matsumura F. Sequences of cDNAs encoding calmodulin, and partial structures of calmodulin kinase, and a calcium channel of *kdr*–resistant and susceptible German cockroaches, *Blattella germanica*. Comp Biochem Physiol C Pharmacol Toxicol Endocrinol. 1998; 120(2): 225–233. 9827036 10.1016/s0742-8413(98)00044-9

[73] Burkert P, Duch C. Developmental changes of CaMKII localization, activity and function during postembryonic CNS remodelling in *Manduca sexta*. Eur J Neurosci. 2006; 23(2): 335–349. 16420442 10.1111/j.1460-9568.2005.04562.x

[74] Lohr C, Bergstein S, Hirnet D. Developmental distribution of CaM kinase II in the antennal lobe of the sphinx moth *Manduca sexta*. Cell Tissue Res. 2007; 327(1): 189–197. 16896952 10.1007/s00441-006-0249-6

